# Role of the ACE ID and PPARG P12A Polymorphisms in Genetic Susceptibility of Diabetic Nephropathy in a South Indian Population

**DOI:** 10.5812/numonthly.9573

**Published:** 2013-05-26

**Authors:** Lakkakula VKS Bhaskar, Sultana Mahin, Raju Thankabai Ginila, Periyasamy Soundararajan

**Affiliations:** 1 Department of Biomedical Sciences, Sri Ramachandra University, Chennai, India; 2 Department of Nephrology, Sri Ramachandra Medical College and Hospital, Sri Ramachandra University, Chennai, India

**Keywords:** Angiotensin Converting Enzyme 2, PPAR Gamma, Alleles, Diabetic Nephropathies

## Abstract

**Background:**

Diabetic nephropathy (DN) is one of the life-threatening disorders characterized by persistent albuminuria, raised arterial blood pressure, a lowered glomerular filtration rate, and high risk of cardiovascular morbidity and mortality. The vascular genes ACE (Angiotensin-converting enzyme), and PPARG (peroxisome proliferator activated receptor gamma) are involved in alterations in vascular endothelium, and are suggested to play a role in the susceptibility of diabetic nephropathy.

**Objectives:**

The aim of our study was to find out the role of ACE ID and PPARG P12A polymorphisms in genetic susceptibility of diabetic nephropathy in south Indian population.

**Patients and Methods:**

A total of 54 cases with diabetic nephropathy and 67 control subjects with diabetes were enrolled for our study. DNA was isolated from peripheral blood leucocytes, and genotyped using PCR-electrophoresis (ACE ID) or PCR-RFLP (PPARG P12A) methods.

**Results:**

ACE ID genotypes followed Hardy-Weinberg equilibrium in both cases and controls. But P12A genotypes deviated from Hardy-Weinberg equilibrium in diabetic controls. Chi^2^ test was applied for the analysis of genotypic distributions in genotypic and dominant models. Odds ratios were also calculated. No significant differences in genotype frequencies of ACE ID and PPARG P12A polymorphisms were found on comparing patients with diabetic nephropathy with diabetic controls. The synergistic role of ACE ID* PPARG P12A interaction, did not show any association in patients with diabetic nephropathy when compared to diabetic controls.

**Conclusions:**

In conclusion, the ACE and PPARG genes do not have a key role in conferring risk for diabetic nephropathy.

## 1. Background

Diabetic nephropathy (DN) is one of the life-threatening disorders characterized by persistent albuminuria, raised arterial blood pressure, a lowered glomerular filtration rate, and high risk of cardiovascular morbidity and mortality (1). The prevalence of diabetic retinopathy continues to rise with the duration of hyperglycemia, but the DN develops in only about one third of the patients, irrespective of glycemic control (2). A significant proportion of patients with diabetes do not develop DN despite long-standing severe hyperglycaemia whereas others develop DN even under intensive insulin therapy. This indicates that other factors apart from chronic hyperglycemia per se may equally contributing susceptibility for the development of DN. Although the exact causes of DN are not fully known, the familial aggregation of the DN indicates the involvement of genetic factors in the pathogenesis of DN (3). Several studies of diabetic nephropathy has analyzed the candidate genes that have previously been studied in hypertension, type 2 diabetes mellitus (T2DM), and cardiovascular disease (4).

Angiotensin-converting enzyme (ACE, EC 3.4.15.1) is an exopeptidase which participates in the renin-angiotensin system (RAS), and mediates various physiological functions. Intron 16 of the ACE gene harbors an insertion-deletion (ID) polymorphism (5), and was thought to influence ACE-mediated physiological functions (6). This polymorphism was known to affect the plasma ACE levels (7). The peroxisome proliferator activated receptor gamma (PPARG) is a transcription factor which regulates the transcription and expression of several genes involved in glucose metabolism and adipocyte differentiation (8). PPARG gene spans more than 100 kb of genomic DNA on 3q25, and is composed of 9 exons (9, 10) coding 60kD PPARG protein.

## 2. Objectives

The present study is aimed to evaluate the role of ACE ID and PPARG and polymorphisms in relation to type-2-diabetes induced nephropathy in south Indian population.

## 3. Patients and Methods

### 3.1. Subjects

Individuals for the study were recruited among patients visiting Sri Ramachandra Medical Centre, Chennai. About 67 unrelated individuals with diabetes for more than 10 years who have not developed nephropathy were considered as controls. 54 individuals with type 2 diabetes and nephropathy were considered as cases. All participants underwent detailed clinical evaluation, and followed by biochemical investigations such as serum creatinine, fasting and postprandial blood glucose, and 24 h urinary albumin excretion. Patients with persistent urine albuminuria (> 300 mg/L) in two consecutive measurements with or without renal failure (serum creatinine > 1 mg/dL) were considered as cases with diabetic nephropathy. All the patients participated in the study had given informed written consent prior to the study. This study was approved by Sri Ramachandra University Review Committee for Protection of Research Risks to Humans. 3 mL of blood sample was collected into EDTA coated vacutainers from all the participants.

### 3.2. Genotyping

The genotypes were determined for each individual by finding the polymorphic combination for the 2 candidate polymorphisms from the ACE and PPARG genes. Genomic DNA was isolated from lymphocytes by standard techniques, and we used published methods to genotype the ACE (11) and P12A (12). The ID polymorphism of ACE was genotyped by using PCR-electrophoresis. To avoid the mistyping of ID genotype as DD, an additional PCR was performed in all the DD samples (13). Single band products with the size of 190 and 490 bp were considered as homozygous DD and II genotypes respectively; whereas, two bands of 190 and 490 bp confirmed heterozygous ID genotype. The P12A was genotyped by amplifying a 156 bp region of exon 2 by using forward primer 5'-ACTCTGGGAGATTCTCCTATTGGC-3', and reverse primer 5'-CTGGAAGACAAACTACAAGAG-3' followed by the digestion of PCR product with HaeIII enzyme. The A12 allele was characterized by the presence of a 156 bp fragment, which was digested further into 133 bp and 23 bp fragments in P12 allele carriers.

### 3.3. Statistical Analysis

The genotype distribution for each polymorphism was evaluated for Hardy-Weinberg equilibrium by using HWSIM program (14). The strength of the association between ACE ID and PPARG P12A genotypes and their interaction in causing the diabetic nephropathy was performed using chi^2^ analysis. For the computation of percentages, odds ratios (OR) with 95% confidence interval and chi square tests were used. We used the statistical package SPSS 14.0 for the analyses.

## 4. Results

The genotypic frequency of ACE ID and PPARG P12A in patients with DN and diabetic controls was presented in Table 1. Of the 54 patients with DN analyzed for ACE ID, 14 (25.9%) were II, 29 (33.7%) were ID, and 11 (20.37%) were DD. Among 67 diabetic controls, 24 (35.82%) were II, 30 (44.78%) were ID, and 13 (19.4%) were DD. The frequency of deleted allele (minor allele) was 47.2% in DN group, and 41.5% in diabetic controls. Thus the frequency of minor allele was almost similar in both diabetic nephropathy and diabetic controls. The expected genotype frequencies were calculated for both DN and diabetic controls, and Hardy-Weinberg equilibrium was calculated based on the goodness-of-fit X ^2^ statistics. The genotype frequencies were in Hardy-Weinberg equilibrium in DN (P = 0.57) and diabetic controls (P = 0.514). The frequency of ID genotype was slightly higher in both diabetic controls than DN cases with an OR (95% CI) of 1.27 (0.52-3.13). The mutant homozygous genotypes were almost similar in both case and control groups, and not associated with DN. Similar trend was observed when analyzed in the dominant model.

For PPARG P12A polymorphism, of the 54 patients with DN analyzed, 37 (68.5%) were P12/P12, 17 (31.5%) were P12/A12, and no A12/A12 were found. Among 67 diabetic controls, 36 (53.73%) were P12/P12, 31 (46.26%) were P12/A12, and 0 (0%) were A12/A12. The frequency of A12 allele was 15.7% in DN group, and 23.1% in diabetic controls. Thus the frequency of A12 allele was slightly less in DN than the diabetic controls. The genotype frequencies were in Hardy-Weinberg equilibrium in DN (P = 0.17) but not in diabetic controls (P = 0.014). The frequency of P12/A12 genotype was slightly higher in diabetic controls than the cases with diabetic nephropathy with an OR (95% CI) of 0.53 (0.24-1.20). As there was no A12/A12 mutant homozygous genotypes in both case and control groups the computation of OR (95% CI) was not possible. Also the association between diabetic nephropathy and PPARG P12A was not observed in the dominant model (Table 1). The synergistic role of ACE ID*PPARG P12A interaction, did not show any association in patients with DN when compared to the diabetic controls (Table 2).

**Table 1.** Association of PPARG P12A and ACE ID Polymorphisms With Diabetic Nephropathy

	Diabetic Nephropathy		OR ^a^ (95% CI ^a^)	P value
	**Yes**	**No**		
**ACE^a^ Ins/Del**				
**II**	14 (25.93)	24 (35.82)	Reference	
**ID**	29 (53.7)	30 (44.78)	1.27 (0.52-3.13)	0.559
**DD**	11 (20.37)	13 (19.40)	1.45 (0.45-4.67)	0.482
**ID+DD**	40 (74.07)	43 (64.18)	1.59 (0.73-3.5)	0.244
**MAF ^a^**	47.2	41.8		
**HWp ^a^**	0.57	0.514		
**PPARG ^a^ P12A**				
**P12/P12**	37 (68.52)	36 (53.73)	Reference	
**P12/A12**	17 (31.48)	31 (46.27)	0.53 (0.24-1.20)	0.098
**A12/A12**	0 (0.0)	0 (0.0)	-	-
**A12 carriers**	17 (31.48)	31 (46.27)	0.53 (0.24-1.20)	0.098
**MAF (%)**	15.7	23.1		
**HWp**	0.17	0.014		

^a^Abbreviations: ACE, angiotensin-converting enzyme; CI, confidence interval; HWp, Hardy-Weinberg equilibrium p value; PPARG, peroxisome proliferator activated receptor gamma, MAF, minor allele frequency; OR, odds ratio

**Table 2.** Effect of PPARG P12A and ACE ID Genotypes Interaction on Diabetic Nephropathy

PPARG P12A	ACE^a^ID	Cases (%)	Controls (%)	Strata Specific OR ^a^(95%CI^a^); P-value	Interaction OR (95%CI); P-value
**P12/P12**	II	11 (20.4)	14 (20.9)	Reference	
**A12 carriers**	II	3 (5.6)	9 (13.4)	0.42 (0.07-2.3); 0.264	
**P12/P12**	ID+DD	26 (48.1)	23 (34.3)	Reference	
**A12 carriers^b^**	ID+DD	14 (25.9)	21 (31.3)	0.59 (0.22-1.5); 0.237	0.54 (0.24-0.54); 0.164

^a^Abbreviations: ACE, angiotensin-converting enzyme; OR, odds ratio; CI, confidence interval

^b^A12 carriers, P12/A12 + A12/A12

## 5. Discussion

Analysis of 67 diabetic controls and 54 patients with DN for PPARG P12A and ACE ID polymorphisms revealed that both are polymorphic in the study subjects. Chi^2^ analysis did not show any significant association between these polymorphisms and DN both at allele and genotype level. The mutant genotypes of both polymorphisms exhibited differences in microalbumin, but the difference was not significant. Both serum creatinine and protein-creatinine ratio did not show any significant differences between the mutants of the two polymorphisms studied.

The ACE gene is one of the important genes in RAS pathway. Although the data from our study failed to confirm an increased risk for development of DN in T2DM, previous studies from Japan, the USA, and Iran showed association between ACE-DD genotype and/or D-allele and the risk for nephropathy in T2DM (15-18). In contrast, studies from Poland and Germany, did not show any association between the ACE gene polymorphism and nephropathy in individuals with T2DM (19, 20). Contradictory findings that observed in the association between ACE ID and DN in north Indian (21) and south Indian populations (22) denoting the importance of this genetic marker in Indian patients with T2DM (17). A meta-analysis of 47 studies published from 1994 to 2004, demonstrated that the II genotype has reduced the risk of DN compared to the D-allele carriers (23). In fact, the deletion polymorphism is associated with elevated serum and cellular ACE levels (5). The elevated ACE expression increases the plasma angiotensin II level, and promotes podocyte injury which leads to progressive kidney diseases as well as DN (24).

The present study did not show any significant association between PPARG A12 allele and DN and its clinical parameters. The results of the present study are consistent with the results of the study from Han Chinese (25), African-Americans (26) and Turkish populations (27). In contrast, A12 allele conferred protection against diabetic nephropathy in Brazilian patients with type 2 diabetes (28). The Berlin Diabetes Mellitus (BeDiaM) Study also supported the protective effect of the A12 allele against diabetic nephropathy (29). Furthermore, PPARG P12A genotype showed a modest effect and is overshadowed by duration of diabetes and systolic blood pressure in the aboriginal Canadian population (30). Although, the mechanisms by which the PPARG P12A polymorphism contributes to diabetic nephropathy is not yet elucidated completely. Earlier studies have demonstrated that the PPARG A12 allele showed decreased binding affinity to promoters. This consecutively implies that the patients with homozygous for the P12 allele, show increased insulin resistance, which leads to diabetic nephropathy.

In conclusion, the ACE and PPARG genes do not play a key role in conferring risk for diabetic nephropathy. Although the ACE D-allele (38.3 to 56.5%) (21, 22, 31-33) and PPARG P12 allele (7.5 to 11.9%) (34-36) showed striking variations in their frequencies among different ethnic groups in different studies. In the present study these alleles were within the range of reported frequency in India. As this study is limited with less number of cases and controls, the genotypic and allelic differences that observed may not represent a true association. Hence, additional studies considering gene-gene and gene-environment interactions should be investigated to estimate the overall risk of the ACE and PPARG genes in the pathogenesis of diabetic nephropathy.
